# [Corrigendum] Deoxypodophyllotoxin inhibits cell viability and invasion by blocking the PI3K/Akt signaling pathway in human glioblastoma cells

**DOI:** 10.3892/or.2023.8484

**Published:** 2023-01-16

**Authors:** Wei Wang, Wei Gao, Luyang Zhang, Dongyong Zhang, Zilong Zhao, Yijun Bao

Oncol Rep 41: 2453–2463, 2019; DOI: 10.3892/or.2019.7016

Following the publication of the above article, a concerned reader drew to the authors' attention that various pairs of the data panels shown for the Transwell migration assays in Fig. 4A-D on p. 2459 featured overlapping data, such that a number of the panels may have been derived from the same original sources.

The authors have examined their original data, and realize that errors were inadvertently made during the assembly of these figure parts. The authors have reassembled Fig. 4 containing alternative data in Fig. 4A-D, and the revised version of this figure is shown on the next page. Note that the revised data shown for this figure do not affect the overall conclusions reported in the paper. All the authors agree with the publication of this corrigendum, and are grateful to the Editor of *Oncology Reports* for allowing them the opportunity to publish this. They also apologize to the readership for any inconvenience caused.

## Figures and Tables

**Figure 4.**
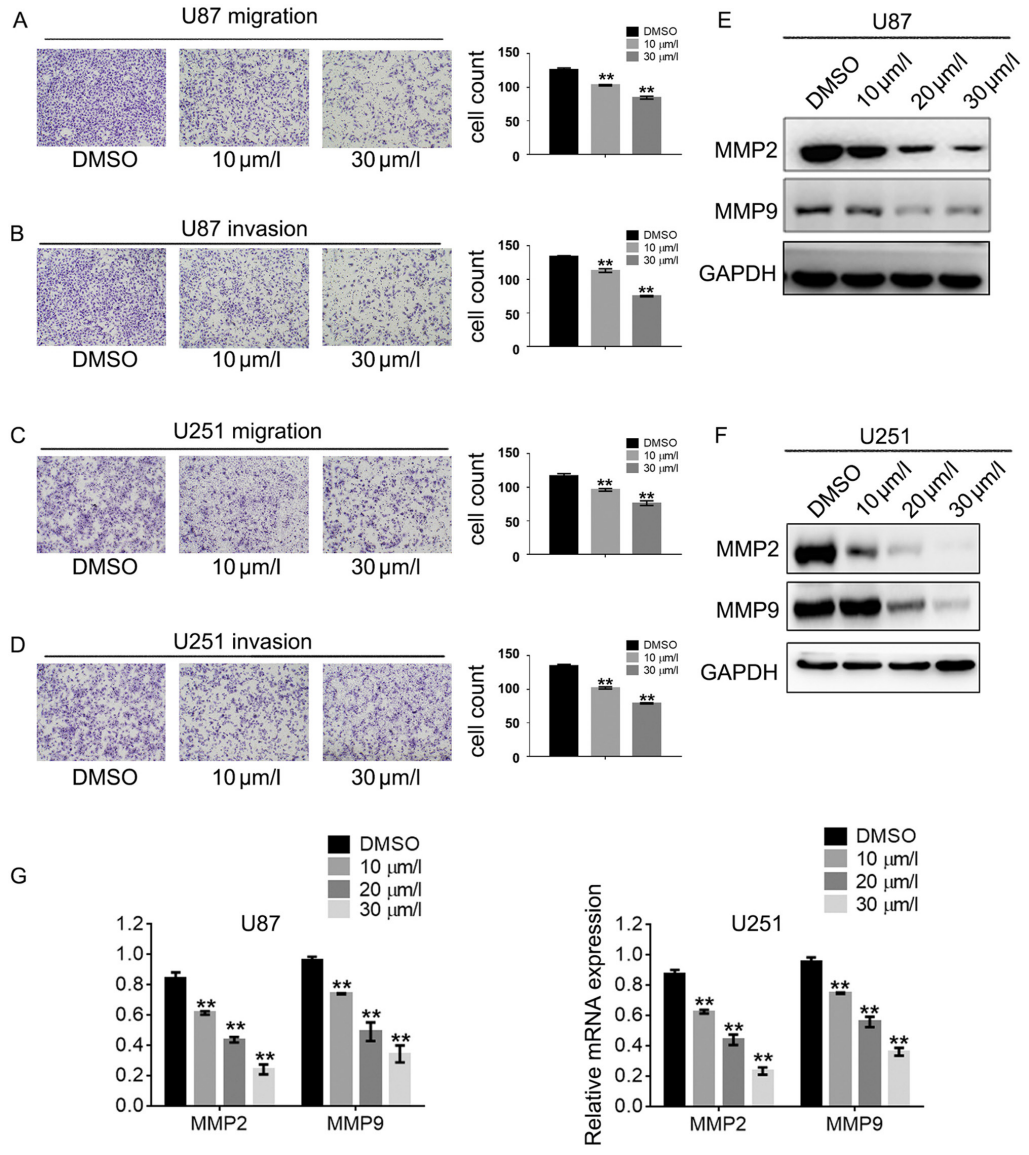
DPT suppresses the invasiveness capacity of GBM cells. U87 and U251 cells were pre-incubated with DPT for 24 h. In U87 cells, (A) migration was assessed by Transwell assay without Matrigel and (B) invasion was assessed by Transwell assay with Matrigel. In U251 cells, (C) migration was assessed by Transwell assay without Matrigel and (D) invasion was assessed by Transwell assay with Matrigel. (E) U87 and (F) U251 cells were treated with indicated concentrations of DPT for 24 h, and the expression of indicated proteins in (E) U87 and (F) U251 cells were detected using western blotting. (G) Relative mRNA levels of MMP2 and MMP9 were detected using reverse transcription-quantitative polymerase chain reaction analysis. Results were presented as the mean ± standard deviation of three experiments performed in triplicate. **P<0.01. MMP, matrix metalloproteinase; DPT, deoxypodophyllotoxin; GBM, glioblastoma; DMSO, dimethyl sulfoxide.

